# Evolving Personalized Therapy for Castration-Resistant Prostate Cancer

**DOI:** 10.7603/s40681-014-0002-5

**Published:** 2014-08-05

**Authors:** Hsin-Ho Liu, Yuh-Shyan Tsai, Chen-Li Lai, Chih-Hsin Tang, Chih-Ho Lai, Hsi-Chin Wu, Jer-Tsong Hsieh, Che-Rei Yang

**Affiliations:** 1 Department of Urology, University of Texas Southwestern Medical Center, Dallas, Texas, USA; 2 Division of Urology, Department of Surgery, Taichung Tzu Chi General Hospital, Taichung, Taiwan; 3 Department of Bio-Industrial Mechatronics Engineering, National Taiwan University, Taipei, Taiwan; 4 Department of Urology, Medical College and Hospital, National Cheng Kung University, Tainan, Taiwan; 5 School of Medicine, China Medical University, Tainan, Taiwan; 6 Department of Urology, China Medical University Hospital, Tainan, Taiwan; 7 Graduate Institute of Cancer Biology, China Medical University, Tainan, Taiwan

**Keywords:** Prostate cancer, Personalized cancer therapy, Castration-resistant prostate cancer

## Abstract

With advances in molecular biologic and genomic technology, detailed molecular mechanisms for development of castration-resistant prostate cancer (CRPC) have surfaced. Metastatic prostate cancer (PCa) no longer represents an end stage, with many emerging therapeutic agents approved as effective in prolonging survival of patients from either pre- or post-docetaxel stage. Given tumor heterogeneity in patients, a one-size-fits-all theory for curative therapy remains questionable. With the support of evidence from continuing clinical trials, each treatment modality has gradually been found suitable for selective best-fit patients: e.g., new androgen synthesis inhibitor arbiraterone, androgen receptor signaling inhibitor enzalutamide, sipuleucel-T immunotherapy, new taxane carbazitaxel, calcium-mimetic radium-223 radiopharmaceutical agent. Moreover, several emerging immunomodulating agents and circulating tumor cell enumeration and analysis showed promise in animal or early phase clinical trials. While the era of personalized therapy for CRPC patients is still in infancy, optimal therapeutic agents and their sequencing loom not far in the future.

## 1. Current therapeutic regimen in prostate cancer

Prostate cancer (PCa) is the lead malignancy among males in Western countries, accounting for 28% (238,590) of newly diagnosed cancers in United States in 2013 [1]. It has been the second common cause of cancer deaths in men (behind lung cancer) for two decades [1, 2]. Treatment for clinically localized PCa aims at cure, typically by surgery or radiation. Emerging technologies have also been used for selected patients in low-risk PCa: e.g., high-intensity focused ultrasound (HIFU), cryotherapy, radiofrequency ablation and photodynamic therapy. For advanced PCa cases, androgen deprivation therapy (ADT) is standard treatment. The majority of advanced PCa patients respond to initial ADT temporarily but inevitably progress from androgen-dependent stage to CRPC. Effective treatment at this stage is largely limited to chemotherapy. Indeed, prior to 2010, only docetaxel chemotherapy shows survival benefit in CRPC. With the most effective standard chemotherapeutic regimens, mean increase in survival time is two months, highlighting the need for more effective treatments [3, 4]

**Figure 1.**
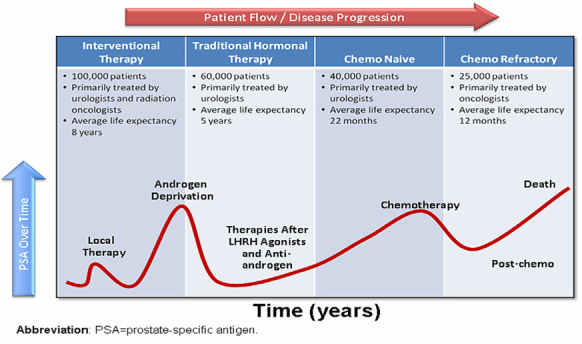
Common clinical course of PCa progression from localized stage to CRPC. PSA level is used as a surrogate for cancer burden; the figure shows PSA rising at the time of initial diagnosis, returning to normal via first-line treatment (radiation or surgery), then rising again as cancer recurs. Again it is reduced by hormonal therapy. When CRPC occurs, PSA again rises and minimally impacted by chemotherapy. After chemotherapy fails, PSA rises until the patient dies. Permission from Dr. Ganesh Raj (Department of Urology, University of Texas Southwestern Medical Center).

Figure 1 plots common clinical course of PCa from localized stage to CRPC. Interpretation of castration resistance pathway could lead to identification of new pathway-targeted therapeutics. Incidence and mortality rates vary widely across geographic regions and ethnic groups [5]. Of note, Asians have substantially lower prevalence than African Americans and Caucasians, indicating linkage between genetic background and susceptibility [6]. Exact molecular mechanisms of prostate carcinogenesis are not fully elucidated, but it is evident that genetic factors at both germline and somatic levels play key roles in carcinogenesis. It has been increasingly recognized that cancer cells are heterogeneous within the same lesion at both genetic and epigenetic levels, which could translate into functional heterogeneity: e.g., self-renewal properties, tumor-initiating ability [7]. Significant tumor heterogeneity appears within primary and metastatic tumor lesions as well as individual cases, challenging standard approach to cancer management and highlighting the need for personalized cancer therapy.

## 2 New strategies in CRPC therapy

In the case of advanced PCa, ADT is standard treatment, which initially reduced tumor burden and prostate-specific antigen (PSA) level to low or undetectable level. Most PCa ultimately recurs despite of ADT, presenting with progressively rising of PSA level, termed CRPC. Docetaxel was regarded as the only reasonable option before 2010. Additionally, there is no therapeutic agent for patients who experience progression after first-line docetaxel. Recent years have seen a number of novel anticancer drugs for CRPC clinics. The past three years can be considered exceptional due to positive outcomes in Phase III trials. Key antitumor agents showing positive results include taxane cabazitaxel [8, 9], vaccine sipuleucel-T [10], cytochrome p450 17 (CYP17) inhibitor abiraterone [11, 12], androgen-receptor antagonist enzalutamide (formerly known as MDV-3100) [13-15], and radioisotope alpharadin (radium 223) [16]. Other promising agents including denosumab [17], orteronel [18], ipilimumab [19] and cabozantinib [20, 21] are currently under study. These novel agents are appropriately applied to the CRPC treatment pathway to maximize therapeutic efficacy.

Cabazitaxel, a second-generation taxane, demonstrably improves overall survival when added to prednisone versus mitoxantrone plus prednisone in TROPIC (treatment of hormone-refractory metastatic PCa previously treated with docetaxel-containing regimen) trial: median overall survival is 15.1 months versus 12.7 months in CRPC patients with progression after docetaxel treatment [8]. Progression-free survival also improves in the cabazitaxel-prednisone treatment arm.

Sipuleucel-T, an active cellular immunotherapy, is a type of therapeutic cancer vaccine consisting of autologous peripheral-blood mononuclear cells (PBMCs), including antigen-presenting cells (APCs) activated *ex vivo* with a recombinant fusion protein (PA2024) [10]. PA2024 consists of a prostate-specific acid phosphatase (PAP) fused with granulocyte-macrophage colony-stimulating factor (GM-CSF), an immune-cell activator. This regimen can reduce death risk by 22%, representing a 4.1-month improvement in median survival [10]. In conclusion, sipuleucel-T prolonged overall survival among asymptomatic metastatic CRPC (mCRPC) patients. Adverse events are more frequently reported in the sipuleucel-T group, including chills, fever, and headache with mainly Grade 1 or 2 in severity.

Abiraterone acetate blocks androgen biosynthesis by inhibiting 17α-hydroxylase/C17,20-lyase (CYP17). The COU-AA-301 and COU-AA-302 trials established the role of abiraterone in mCRPC patients with or without previous docetaxel chemotherapy. In COU-AA-301 trial, overall survival as primary endpoint was longer with abiraterone acetate-prednisone than with placebo-prednisone (14.8 vs. 10.9 months; P<0.001) [11]. In COU-AA-302 trial, radiographic progression-free survival was also longer with abiraterone-prednisone group than with prednisone alone (16.5 vs. 8.3 months; P<l0.001) [12]. Hence abiraterone acetate significantly prolongs overall survival of mCRPC patients, with or without previous docetaxel chemotherapy.

Enzalutamide, a novel androgen receptor signaling inhibitor, competitively inhibits binding of androgens to the androgen receptor (AR), inhibits AR nuclear translocation, and inhibits association of the AR with DNA [22]. The AFFIRM trail (A multinational phase 3, randomized double-blind, placebo-controlled efficacy and safety study of oral MDV3100 in progressive CRPC previously treated with docetaxel-based chemotherapy) confirms that enzalutamide could benefit men with post-docetaxel CRPC [15]. Enzalutamide is well-tolerated and prolongs overall survival with median survival of 18.4 months, slows disease progression, and improves quality of life in men with post-docetaxel CRPC. It reduces risk of death by 37% relative to placebo [14, 15].

**Table 1.** Novel strategies for CRPC therapy

Category	Mechanism/ Drug	Reference
Taxane	Inhibits microtubule depolymerization	
	Docetaxel	[3,4]
	Cabazitaxel	[8,9]
Immunotherapy	Autologous immunotherapy Sipuleucel-T	[10,65,66,67]
	Immune checkpoint inhibitor	
	Ipilimumab	[69]
	Tremelimumab	[70]
AR signaling inhibitor	Androgen receptor antagonist Enzalutamide	[14,15]
	CYP17 inhibitor Abiraterone acetate	[11,12,41]
	Orteronel	[46,47]
	Galeterone	[46,47]
	VT-464	[47]
	HSP90 chaperone inhibitors Geldanamycin	[44]
	Histone deactylase inhibitors Vorinostat (SAHA)	[44]
Tyrosine kinase inhibitor	Against MET and VEGFR2 Cabozantinib	[20,21]
PI3K pathway inhibitor	PI3K Inhibitors	[36]
	XL147	
	BEZ235	
	GDC-0941	
	AKT inhibitors	[36]
	GSK690693	
	MK2206	
	mTOR inhibitors	[36]
Alpha-pharmaceuticals	Irradiation causes double-strand DNA break Alpharadin	[16]

**Figure 2.**
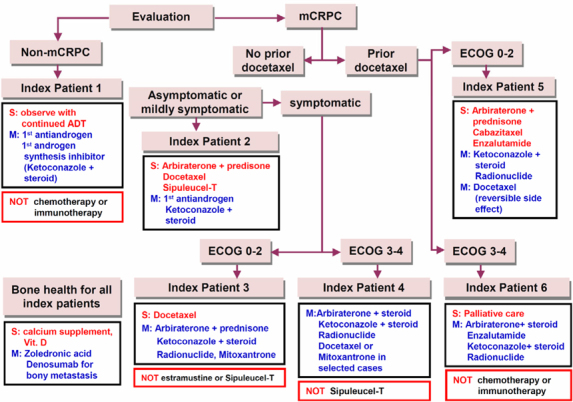
Modified version of the algorithm of American Urologic al Association guideline that represents personalized therapy prototype for CRPC (adapted from [25]). S: suggesting treatment for therapeutic agents; M: considering treatment for therapeutic agents; Black rectangle: suggesting treatment for categorized therapeutic agents; Red rectangle: not recommending treatment.

Radium-223 (alpharadin), calcium-mimetic radiopharmaceutical, has high bone affinity. Alsympca (ALpharadin in SYMptomatic Prostate Cancer) Phase III trial shows improved overall survival: median duration 14 months [16]. Time to first skeletal-related event (SRE) also improves, with median duration of 13.6 months.

Cabozantinib (XL184), an orally bioavailable tyrosine kinase inhibitor, shows potent activity against MET and VEGF Receptor 2 (VEGFR2). It suppresses MET and VEGFR2 signaling, rapidly inducing apoptosis of endothelial and tumor cells, resulting in tumor regression. It can also block progression of osteolytic and osteoblastic lesions [20, 21]

## 3. Personalized therapy

It is well documented that response to standard therapy differs among patients diagnosed with the same cancer. Obviously, a one-size-fits-all concept is not expected to achieve identical outcome; individualized approach is needed. Progress in understanding intricate molecular mechanisms for transformation of normal cells into cancer, plus aberrant control of complementary pathways, leads us into a more complex world for diagnosis and treatment. Oncology has entered an era with treatment individualized or customized, therapy based on molecular and genetic traits of a tumor and its microenvironment, tailored to improve outcomes and decrease both toxicity and health-care costs. Personalized cancer therapy targets aberrations that drive tumor progression, administering the right therapy for the right person at the right time. Success requires identification of novel validated markers for prognosis, treatment response, resistance and toxicity. Chief task in practice is modifying therapy for diverse tumor nature with inadequate, limited prognostic tools [23, 24].

The American Urological Association (AUA) announced clinical guidelines for CRPC in May, 2013 [25]. With several Food and Drug Administration (FDA)-approved therapeutic agents for mCRPC debuting over the past three years, urologists and other clinicians face challenges with multiple treatment options. Potential sequencing of these agents further makes clinical decision-making more complex than ever. To assist in clinical decision-making, AUA developed six index patients to represent the most commonly encountered in clinical practice, which based on the presence or absence of metastatic disease, the degree of symptoms, the patients’ performance status, and the prior docetaxel-based chemotherapy. Such guidelines constitute a prototype of personalized therapy for CRPC (Figure 2).

## 4. Genomic strategy for targeting therapy in CRPC

Large-scale cancer genomic characterization projects offer critical new insights into molecular classification of cancers and have potential to identify new therapeutic targets [26]. PCa exhibits heterogeneous epidemiological and clinical aspects, likely a reflection of underlying genomic diversity. From a molecular viewpoint, cancer can result from a combination of single nucleotide variants (SNVs), small insertions and deletions (indels), chromosomal rearrangements, aberrant DNA methylation and copy number alterations (CNAs), which engenders different expressions of oncogenes or tumor suppressors. In the long run, gathering the entire genomic and transcriptomic landscape of PCa, as well as defining frequency of alteration in several common signal transduction pathways, can further correlate genomic alterations to clinical outcome.

### 4.1 Copy number and transcriptome profiles define core pathway alterations

Copy number alterations (CNAs) can result in amplification of oncogenes or deletion of tumor suppressor genes; these changes contribute significantly to cancer etiology. Consistent and common findings from global analyses of CNAs within PCa include *TMPRSS2-ERG* fusion (around 50%) [27, 28], 8p loss (30-50%) and 8q gain (20-40%) [29, 30]. Focal amplifications of *AR* (Xq12) and *MYC* (8q24), and homozygous focal deletions of *PTEN* (10q23) and *NKX3.1* (8p21) are frequently identified in PCa [31, 32]. Recent CNA study of 218 primary and metastatic tumors added a key role for somatic copy number increases of *NCOA2* gene, which encodes an AR coactivator [30]. In detail, besides above descriptions, peaks of deletion targeting *RB1* on 13q14.2, *TP53* on 17p31.1, interstitial 21q22.2-3 deletion spanning *ERG* and *TMPRSS2* [30], deletions on 12p13.31-p12.3, which spans *ETV6* and *DUSP16* as well as *CDKN1B* [29] were reported. Most common amplified loci include *MYC* on 8q24.21 and *NCOA2* on 8q13.3. Focal amplification of *AR* (Xq12) is likewise common but restricted to metastatic tumors. Among mutated genes, the most common is androgen receptor (*AR*); other oncogenes like *IDH1, IDH2, PIK3CA, KRAS*, and BRAF do not commonly mutate in PCa [30]. There is no great correlation between histology (Gleason score) and CNAs; the latter could serve as an independent clinical marker from Gleason score [33]. Integrating CNAs, transcriptome, and mutation data can further conduct core pathway analysis for PCa. Three recognized cancer pathways, PI3K, RAS/RAF, and RB, are ordinarily altered in primary PCa (range: 34-43%) and metastases (74-100%). Of particular interest is PI3K pathway, altered in nearly half the primaries and all metastases examined [30]. Loss of PTEN function is well documented in PCa: estimated frequency around 40% [34]. PTEN negatively regulates PI3K/Akt pathway; loss of PTEN activity may lead to permanent PI3K/Akt activation. Frequency of PI3K pathway alteration rises substantially when *PTEN* alteration is considered with *INPP4B* and *PHLPP* phosphatase alterations recently implicated in PI3K regulation, the *PIK3CA* gene itself, and regulatory subunits *PIK3R1* and *PIK3R3* [26]. Exploring novel PI3K pathway inhibitors may reap therapeutic benefit [35, 36].

### 4.2. Genetic alterations highly associated with TMPRSS2-ERG

A recent rearrangement involving the androgen-regulated *TMPRSS2* and members of the ETS transcription factor family (*ERG, ETV1, ETV4*) has been identified in a majority of prostate cancers [27, 37]. Further functional studies of *TMPRSS2-ERG* have shown modest evidence of oncogenic activity with cooperating transforming events [27, 28]: *TMPRSS2-ERG* fusion as the single most established PCa molecular lesion [27], meaning expression of N-terminally truncated ERG protein under control of *TMPRSS2* androgen-responsive promoter [38]. Significant regions of copy-number loss link with *TMPRSS2-ERG* fusion: spanning tumor suppressors *PTEN* and *TP53*, plus another spanning 3p14 multigenic region. The 3p14 deletion, whose association with *TMPRSS2-ERG* loomed predominant, appeared only in PCa [30, 39]. Homogeneous distribution of *TMPRSS2-ERG* fusion in 19% of high-grade prostatic intraepithelial neoplasia (PIN) lesions and in 50% of localized PCa suggests this fusion as either occurring after onset or associated with early events predisposing to clinical progression [38]. Recent genomic studies show how ERG binds to AR-regulated genes and alters AR signaling in PCa cells via epigenetic silencing, invariable with a role in inhibiting prostate epithelial differentiation and turning on EZH2 expression, which initiates stem cell-like de-differentiation and carcinogenesis [40]. Population-based studies hint ETS fusion-positive cancer as aggressive in nature and support early detection-based efforts. Commercially available urine test for *TMPRSS2-ERG* is technically feasible nowadays; in PSA-screened cohorts it shows sensitivity of 30-50% and specificity >90%. Examination for *TMPRSS2-ERG* may detect 15-20% of men harboring PCa but with normal DRE (digital rectal examination) and PSA levels, including a substantial proportion of those who harbor high-grade Gleason disease [41]. Most 5’ end ETS fusion partners are androgen responsive; targeting androgen signals may act at least in part by inhibition of ETS fusion. Recent studies indicated a highly specific CYP17 inhibitor, abiraterone acetate, ablating androgen and estrogen syntheses that drive *TMPRSS2-ERG* fusions, inducing regression in >50% of CRPC cases [42]. Hormone-dependent overexpression of ERG persisted in CRPC, and *TMPRSS2-ERG* tumors manifested a subgroup of PCa remaining exquisitely sensitive to CYP17 blockade [43]. Also, ETS gene-fusion status may serve as a prospective character of androgen dependence in CRPC state [44]. As deregulated transcription factors, ETS fusions may drive PCa via induction of downstream target genes, maybe offering a target as therapeutic strategy.

### 4.3 Androgen receptor (AR) signaling pathway

AR signaling is essential for growth and differentiation of a normal prostate and is responsible for treatment failure in CRPC or metastatic PCa. The contribution of AR to prostate tumorigenesis and disease progression is incontrovertible. The exclusive requirement of PCa cells for AR activity is illuminated at clinic, wherein therapeutic suppression of AR signaling, typically achieved through ligand depletion and direct AR antagonists, results in PSA decline and objective tumor regressions. Conventional therapy currently focuses on androgen-dependent activation of AR via its C-terminal ligand-binding domain (LBD). Mechanisms of therapeutic failure include AR amplification and/or overexpression, gain-of-function AR mutations, intracrine androgen production; overexpression of AR coactivators, expression of constitutively active splice variants of AR, and ligand-independent AR activation through growth factors, cytokines, or aberrant AR phosphorylation [45]. Among AR pathway genes, the most prominent finding is a peak of copy-number gain on 8q13.3 that spans the nuclear receptor coactivator gene *NCOA2* [30]. High frequency of *NCOA2* gain in primary tumors plus a known role as AR coactivator [46] lends insight into how these two genes collaborate in early PCa progression by enhancing AR transcriptional output. *NCOA2* functions as a driver oncogene in primary tumors by increasing AR signaling; in contrast, AR amplification is largely restricted to mCRPC and likely a mechanism of drug resistance rather than a natural step in tumor progression.

Recently developed androgen-ablative and AR antagonist strategies that achieve complete androgen ablation and sufficient suppression of AR signaling in the prostate improve efficacy of AR targeting and subsequent therapeutic outcome. A new means to deplete androgens is a selective CYP17 inhibitor, which inhibits both testicular-derived androgen production and tumor-derived androgen synthesis, meaning a great advance toward durable androgen depletion and suppression of AR activity. Despite strong rationale for aiming at CYP17, this target remains largely unexploited, with relatively few candidate agents progressing to clinical trials and only ketoconazole, an unspecific CYP17 inhibitor, in widespread clinical use [47]. Promising clinical results from abiraterone acetate in CRPC cases have recently been reported [11]; its efficacy has spawned clinical development of other androgen biosynthesis inhibitors. Orteronel (TAK-700), oral non-steroidal imidazole CYP17 inhibitor, is reportedly more selective for 17,20 lyase activity than abiraterone acetate, but according to recent data from Phase III clinical trial of ortoronel plus prednisone in treatment of progressive mCRPC, ortoronel plus prednisone would not demonstrate a pre-specified level of clinical efficacy. While ortoronel never met the primary endpoint of improved OS (HR=0.894, p=0.226), it did show advantage as secondary endpoint of radiographic progression-free survival (HR=0.755, p<0.001) and posed no major safety concern. Galeterone (VN/124-1, TOK-001), an oral agent, functions both as CYP17A1 inhibitor and anti-androgen, causing AR protein degradation. Preclinical data averred that galeterone may represent the next generation of therapy for cases of CRPC and disease that has progressed despite treatment with enzalutamide. Phase III trails for galeterone are expected in the near future. VT-464, non-steroidal small molecular 17,20 lyase inhibitor, is also in early-phase testing for men with CRPC [48].

Direct AR antagonists are often combined with orchiectomy or GnRH agonists/antagonists, to inhibit AR signaling further. Docking of AR antagonists into the AR C-terminal LBD results in both passive AR inhibition, via competition for agonists, and active mechanism of AR inhibition: e.g., prevention of coactivator binding and inducement of corepressor recruitment. AR can be alternatively spliced so that the C-terminal domain is deleted, rendering AR constitutively active [49]. Splice variants are refractory to traditional androgen deprivation and AR antagonists, highlight that the new class of AR-inhibitory agents must be developed for successful management of tumors expressing truncated AR, wherein even total androgen ablation has no effect on receptor activity. Options for suppressing function of C-terminal-deficient ARs already exist. HSP90 inhibitors (geldanamycin) and agents modulating HSP90-histone deacetylase interactions (genistein) both show capacity for reducing overall AR levels as well as suppressing action of both full-length and truncated AR [45]. Several studies implicate AR N-terminal domain (NTD) as key mediator of ligand-independent AR activity in PCa cell. Alternative means to inhibit AR function by using a decoy molecule representing AR NTD demonstrably suppress tumor growth and hormonal progression [50]. Intratumor injection of lentivirus expressing AR NTD decoy fragment inhibited growth of established LNCaP xenografts [51]. Development of shorter decoy peptides to AR NTD means great challenges: e.g., how to retain both specificity for AR and antitumor activity, maintain peptide lability and requirement of nonlinear regions of AR NTD needed for protein-protein interactions. Lastly, it has been recently shown that AR may require histone deacetylases (HDACs) for transcriptional activation; HDAC inhibitors cooperate with AR-directed therapeutics to enhance cellular response. Novel understanding of AR function during disease progression has scored breakthroughs in novel AR antagonists and ligand-depletion strategies. Stratification of CRPC patients according to disparate AR reactivation may reap the greatest benefit: e.g., for recurrence associated with AR mutations or splice variants inducing resistance to AR antagonists, it is unlikely that the latter would help. This advance is expected to provide new insight into CRPC mechanisms, serving as a base for personalized medicine.

### 4.4. Epigenetic alterations

Epigenetics is defined as heritable changes in gene expression caused by mechanisms other than altered DNA sequence. Unlike many other genetic changes, epigenetic processes are reversible and do not change DNA sequence or quantity, though they enhance genomic instability that might lead to oncogenic activation and inactivation of tumor suppressors [52]. Among types of epigenetic change, the most crucial are DNA methylation and histone modification, both prominent in cancer progression. DNA methylation causes gene-silencing either by inhibiting access of target binding sites to transcriptional activators and/or by promoting binding of methyl-binding domain proteins, which interact with HDACs that promote chromatin condensation into transcriptionally repressive conformations. DNA methylation is thought to alter chromosome structure and define regions for transcriptional regulation. Covalent modification of multiple DNA sites by methylation is heritable and reversible, involved in regulating a gamut of biological processes

[53]. Several classes of drugs, including inhibitors of DNA methyltransferases and HDACs, are known to modify epigenetic information in a fashion not specific to genes. AR may require HDACs for transcriptional activation; HDAC inhibitors may cooperate with AR-directed therapeutics to elicit enhanced cellular response. HDAC inhibitors show promise as therapeutic targets with potential to reverse aberrant epigenetic states associated with PCa.

## 5. Personalizing treatments with circulating tumor cells (CTCs)

CTCs appear in the bloodstream, having detached from their tumor of origin. A major cause of cancer-associated mortality is tumor metastasis, which depends on successful dissemination to the whole body, mainly through blood. Therefore, CTCs shed into vasculature and possibly on the way to potential metastatic sites arouse obvious interest. Studies in past years have shown CTCs as markers predicting cancer progression and survival in metastatic [54-57] or even early-stage cancer patients [58]. Assessment CTC using CellSearch has been cleared by the FDA as a prognostic indicator for patients with metastatic breast, prostate, and colorectal cancers [54, 59]. Increasing CTC numbers correlate with aggressive disease, increased metastasis, and decreased time to relapse in CRPC [55, 60, 61]. CTCs could serve as a real-time monitor for progression and marker for survival and thus have potential to guide therapeutic management, indicate therapy effectiveness or necessity, even while metastases are still undetectable, and offer insights into mechanisms of drug resistance. Thus, CTCs not only could be used as a surrogate endpoint marker in clinical trials [62], but also could become a treatment target [63]. Discrepancy in gene expression between primary tumors and CTCs, as well as heterogeneity within the CTC population, can be observed frequently. To such a degree, it is possible to identify their tissue of origin via expression profiling to detect organ-specific metastatic signatures. This could help to localize small metastatic lesions and afford valuable insight into further diagnostic and therapeutic strategies [64].

Although CTC counts are of prognostic relevance, CTC enumeration is not yet validated as a surrogate of clinical benefit. Technical challenge in this field consists of finding tenuous tumor cells (a few CTCs mixed with approximately 10 million leukocytes and 5 billion erythrocytes in 1 ml of blood) and distinguishing them from epithelial non-tumor cells and leukocytes. It should be feasible with advanced technology that allows automated and high-throughput separation, visualization and quantification of cancer cells from blood [59]. Ability to evaluate longitudinally gene amplifications, mutations, deletions or translocations playing crucial roles in CRPC pathogenesis with CTCs lends unique insight into underlying and evolving biology of tumor, without need for invasive biopsies [65]. This will also allow analysis of molecular changes that occur secondary to treatment pressures and intra-patient tumor heterogeneity that may otherwise have been missed with tumor biopsies. It also allows patient sub-classification according to molecular profiles of risk, prognosis and likely response [66]. Molecular characterization of CTCs may lend insight into underlying mechanisms of resistance to cancer therapy and develop biomarkers to support rational molecular stratification of patients with CRPC to novel antitumor agents. Ultimately, deep sequencing of DNA from CTCs will permit detection of tumor heterogeneity of CRPC, in order to dissect clonal evolution and aid understanding of clones’ association with drug resistance. Future research on CTC enumeration may pinpoint a robust biomarker with strong statistical association to clinical benefit from treatment, which may be employed as a surrogate for true outcome in patients with CRPC. Moreover, CTCs may expedite anticancer drug design, minimizing delay in development and regulatory approval of effective agents for CRPC, reducing the number of patients undergoing ineffective regimen.

## 6. Personalized immunotherapy for PCa

The concept of immune modulation, which aims at generating a meaningful antitumor immune response, has been extensively evaluated in melanoma and renal cell carcinoma. This principle has been extended to PCa, known as slow-growing and more indolent, which can allow sufficient time for generating effective antitumor immune response. Moreover, recent studies indicated that PCa is more immunogenic than considered earlier, with evidence of PCa-specific autoantibodies in blood samples of patients. Sipuleucel-T is the first immunotherapy approved by the US FDA in April 2010 [10]. It is indicated for treatment of asymptomatic or minimally symptomatic mCRPC based on IMPACT (Immunotherapy for Prostate AdenoCarcinoma Treatment) trial, making it the first of its kind vaccine therapy approved for advanced solid tumors. Sipuleucel-T is active cellular cancer vaccine, stimulating immune response to PCa. First, leukopheresis is followed by enrichment of PBMCs, which are incubated with targeted immunogen PA2024, a PAP recombinant fusion protein, and GM-CSF before intravenous administration. Once infused, autologous PBMCs are thought to mature into functional APCs, and activate PAP-specific CD4+ and CD8+ T cells. These activated T cells then home in on tumor lesions, mediating an antitumor response [67-69]. The IMPACT trial, a Phase III, randomized, double-blind, placebo-controlled study, enrolled 512 patients with asymptomatic or minimally symptomatic mCRPC without visceral metastases. Patients were assigned in a 2:1 ratio to receive sipuleucel-T or placebo administered intravenously every two weeks for total three infusions. The primary and secondary endpoints in this study were median overall survival and time to objective disease progression. The sipuleucel-T group had a relative reduction of 22% in risk of death as compared with the placebo group, representing a 4.1-month improvement in median survival (25.8 vs. 21.7 months); 36-month survival probability was 31.7% in the sipuleucel-T versus 23.0% in the placebo group. But secondary endpoint, time to objective disease progression, was not met, similar in both groups. Immune responses to the immunizing antigen were observed in patients receiving sipuleucel-T. Adverse events such as chills, fever and headache were more frequently reported in the sipuleucel-T than in the placebo group [10]. Recent studies in tumor immunology have also focused on the concept of immune checkpoints, a series of molecules that function to limit an ongoing immune response [68, 70]. The ability of cancer cells to evade anti-tumor T-cell activity in microenvironment has recently been accepted as a hallmark of cancer progression. Blocking of one or more such immune checkpoints with monoclonal antibodies (mAbs) has been shown to rescue otherwise exhausted antitumor T cells. Blocking checkpoints to recover existing antitumor immune responses might presumably be more effective than inducing a *de novo* antitumor response through vaccination. Both pilimumab (MDX-010) and tremelimumab (CP-675206) are clinical applications of checkpoint inhibitors, antibodies specific for cytotoxic T lymphocyte antigen 4 (CTLA-4). Ipilimumab is an antagonistic mAbs that recognizes CTLA-4, an immunomodulatory molecule expressed by activated T cells, and to CD80 on APCs. It was proven active both in PSA response and clinical improvement, with or without radiotherapy in mCRPC patients [71]. Because anti-CTLA-4 mAbs target the immune system instead of the tumor, they hold potential advantages over traditional antitumor mAbs, chemotherapy, and immunotherapy (vaccines and cytokines). Other antibodies with neutralizing function, such as CD137, CD40, and PD-1 (programmed cell death 1), are currently in various stages of preclinical and clinical evaluation.

Most treatment regimens for advanced cancer highlight a combination of chemotherapy drugs, or concurrent radio-chemotherapy, raising a possibility that immunotherapy may need combination with conventional therapy to achieve maximal effect. Fortunately, conventional cancer treatments have immunological benefits [72], making combinatorial trials attractive. In sum, there is strong rationale for combined immunotherapies and/or combining immunotherapy with conventional therapy, but such combination increases complexity for clinical trial design; issues of dosing and sequence become a great challenge.

## 7. Conclusions and perspectives

In the past decade, cancer therapy has slowly but steadily transformed from a one-size-fits-all to a more personalized approach, each patient treated according to specific genetic defects of his/her own tumor. Appearance of genomic technologies has now provided the means to develop data that address complexity of biologic states. Practice of cancer therapy continually faces the challenge of matching the right therapeutic regimen with the right patient at the right time, balancing relative benefit with risk to attain optimal outcome. Cases with CRPC may represent myriad heterogeneity in terms of performance, comorbidity, and underlying molecular mechanisms. Prior to 2010, the sole agent for CRPC was docetaxel; positive results now available from clinical trials of cabazitaxel, sipuleucel-T, abiraterone, and enzalutamide mean we now have a plethora of agents to choose from. New AUA guidelines for CRPC treatment in 2013 represent a prototype of personalized therapy. Despite these recent advances, efforts in molecular therapeutics should continue and bring further changes in the PCa treatment paradigm. Moreover, many emerging personalized therapies are under scrutiny: e.g., immunotherapy and CTC-targeted therapy. Though personalized therapy for CRPC is still in its infancy, ideal therapy tailored for individual CRPC patients continues to advance.

## Acknowledgments

We thank Dr. Ming-Chei Maa and Dr. Yuan-Man Hsu for valuable suggestions and editorial assistance. The authors thank Dr. Ganesh Raj (Department of Urology, University of Texas Southwestern Medical Center) for permission to use Figure 1 in this review. This work was funded by the National Science Council (NSC101-2314-B-006-011- MY3 and NSC101-2313-B-039-004-MY3), China Medical University (CMU102-S-28), and the Tomorrow Medicine Foundation.
